# “Like a nurse but not a nurse”: Clinical Research Practitioners and the evolution of the clinical research delivery workforce in the NHS

**DOI:** 10.1186/s12961-019-0462-x

**Published:** 2019-06-11

**Authors:** Rachel Faulkner-Gurstein, Helen C. Jones, Christopher McKevitt

**Affiliations:** 1 School of Population Health and Environmental Sciences, King’s College London, London, United Kingdom; 2 Research and Development Department, Royal Free London NHS FT, London, United Kingdom

**Keywords:** Clinical Research Practitioners, research delivery, research workforce, research capacity

## Abstract

**Background:**

Clinical research is increasing across the United Kingdom. Within the context of a shortage of nurses, trusts have struggled to maintain research capacity. In order to meet staffing demands, trusts have increasingly turned to Clinical Research Practitioners (CRPs) to assist in the delivery of clinical research. Initially an ad hoc workforce, the CRP role is being formalised and professionalised. This study is a close examination of the clinical research delivery workforce at one research-intensive acute trust in the United Kingdom, with a focus on the emerging CRP staff group.

**Methods:**

The study was conducted in a large inner-city teaching hospital (acute trust) in London, United Kingdom. Data were collected between September and December 2017. Twenty-five staff were interviewed across 11 different directorates. Interviews were semi-structured with an overall focus on research staff backgrounds and roles, as well as their perceptions and experiences of careers in research. The data were managed using NVivo 11 software and analysed thematically.

**Results:**

CRPs are drawn from a wider pool of educational and employment backgrounds than their nurse colleagues, and actively seek out work in health research. CRPs receive on-the-job training to acquire the competencies that are necessary for them to carry out their work. The CRP role, which began in an ad hoc manner, has become regularised, yet remains to be well defined, which can be a source of frustration for CRPs and those who work with them.

**Conclusions:**

The professionalisation of the CRP workforce represents an opportunity for the health research system to employ research workers who possess a range of in-demand skills and to shore up research capacity in the context of the shortage of nurses.

## Background

The United Kingdom is in the midst of a golden age for clinical research. Thirteen years on from the establishment of the National Institute for Health Research (NIHR) as the research arm of the National Health Service (NHS), life sciences research has pride of place in the United Kingdom government’s forward-looking Industrial Strategy [1]. The NIHR reports year-on-year increases in the number of patients recruited to research and the number of new studies added to the portfolio. Commercial research is also on the rise and is taking place across the 99% of trusts now active in research [2–4]. However, as the volume of research has increased, some trusts have struggled to meet and maintain research capacity. In this paper, we address research capacity questions through an analysis of an emerging professional role and career within the NHS, that of the clinical research practitioner (CRP).

### Health research delivery

Within the broader process of health research, particular workers within healthcare organisations perform concrete tasks to produce usable data for health research in a process often referred to as ‘research delivery’. For example, Clare Morgan, Research Delivery Director at the NIHR, defines research delivery as “*all the elements, systems and processes and governance that needs to be in place to ensure research is effectively delivered within the NHS*” [Personal communication between Helen Jones and Clare Morgan, 2016]. Building on this conception, we argue that research delivery should be seen as a part of a broader process that includes formulating a research question, developing a protocol and seeking regulatory approval on one side, and data analysis, scientific peer-reviewed publications, and the development of applications and further research questions on the other. Table 1 provides a schematic picture of the research process. The segments referred to as research delivery are highlighted in italics.

**Table 1:** The research process

Question formulation	Protocol development	External approvals process	*Internal approval process*	*Patient recruitment and enrolment*	*Data collection, processing and management*	Analysis	Publication and further development
→	→	→	**→**	**→**	**→**	→	→

Research delivery includes a number of discrete tasks or stages, not all of which will be relevant to every trial or type of research. These tasks might involve providing study information to patients, patient recruitment, screening and consenting, randomisation, undertaking trial assessments, administering study drugs, collecting, processing and managing clinical research data, conducting follow-up visits, engaging in Patient and Public Involvement initiatives, and safety reporting. In addition to the patient-facing aspects of research delivery, where workers have direct contact with patients, many research delivery staff also undertake research management tasks such as overseeing internal Research and Development approval processes, ensuring adherence to regulatory and governance frameworks, managing other research staff, and liaising with support departments. Multiple staff groups are involved in research delivery, including nurses, clinical trials coordinators and practitioners, clinical support staff, data managers, and laboratory, technical and administrative staff. There are also specialists in the form of research delivery managers and directors. Despite their relatively low public profile, clinical research delivery staff, including CRPs, are in fact the largest member group of the NIHR faculty [5] and are vital for the conduct and delivery of clinical research within the NHS.

### The changing health research workforce

In this paper we focus on CRPs, an emerging staff group within the research delivery workforce who are increasingly taking on roles and responsibilities that were once performed primarily by clinical research nurses (CRN). Until recently, most of the patient-facing research delivery workforce had been made up of registered nurses who came to research after spending time working on hospital wards. Increasingly, health research systems now also rely upon CRPs, who are patient-facing staff without formal clinical training and with varied educational backgrounds and experiences. This change has not been examined in depth in the existing literature. Research has focused on the educational backgrounds needed to increase research capacity [6], the availability of research-active investigators to pursue research careers [7, 8], the process of raising research awareness and understanding within healthcare systems [9], and the barriers to the recruitment of patients into trials [10]. In contrast, we seek to fill the gap in the literature by focusing on this new and increasingly important role within research delivery.

In the existing literature on research delivery, the role of CRNs has been well documented. This too is a relatively new role; though nurses worked in research before the NIHR was established, it is only since 2006 that research nursing has emerged as an increasingly professionalised and in-demand specialisation. Early studies on research nurses sought to define the role [11–13] and delineate a scope of practice [14–16]. The Royal College of Nursing has also worked with the NIHR to develop research nurse competencies and career pathways and they jointly released the Clinical Research Nursing Strategies in 2013 [17] and 2017 [18] to increase the visibility of CRNs and further develop their career. Reflecting the maturation of the field, more recent studies have focused on the challenges that research nurses encounter and their impact on the clinical research enterprise. For example, Tinkler et al. [19] detail the dual nature of the research role, highlighting the sometimes-awkward balance of research and caring priorities felt by nurses, and the implications of these conflicting imperatives for professional identity and research delivery.

However, if the rise of the research nurse has been well documented, no comparable literature exists on the CRP. This is perhaps because it is a very new role, having originated alongside increases in funding for research made available through the creation of the NIHR on the one hand [20] and a shortage of nurses within the NHS on the other [21–23]. The lack of literature about CRPs could also reflect inconsistent professional terminology; in the trust where our research took place, an internal staff census conducted in 2017 identified 30 job titles that applied to this group; by comparison, research nurse titles within the same workforce totalled 19, including midwives, matrons and senior managers [data drawn from internal report, full citation hidden to protect anonymity]. The lack of consistent terminology means that, in instances where CRPs are discussed, no standard title is available. Indeed, the only journal article we could find that specifically focuses on this staff group is a United States-based study that refers to them simply as ‘non-nurses’ [24], a label that could be perceived as uninformative and even derogatory. In the study, the authors examine the perspective of nurses on the activities performed by non-nurse clinical research coordinators and do not directly interview any of the ‘non-nurses’ discussed. The lack of a recognised name has thus contributed to the invisibility of the CRP staff group, despite their growing ubiquity within the field of hospital-based clinical research.

As clinical research increasingly includes CRPs in its organisation and delivery, clinical research systems must anticipate and mitigate new categories of risk. Writing about the growing number of non-nurse clinical research coordinators in the United States, Jones et al. warn that “*the delegation of clinical activities typically requiring nursing licensure to unsupervised non-nurses*” can create “*risks to research participants and the quality of clinical research*” [24]. This is not because individual CRPs are not competent to perform the clinical tasks they have been assigned, but rather that, in the absence of a recognised scope of practice underpinned by standardised education and training, there is no agreed-upon measure by which to evaluate their skill or hold them to account. This produces a situation of variability and unaccountability that is at odds with the safe and effective management of clinical trials. Jones et al. [24] argue that elaborated and well-defined role boundaries can help mitigate these risks by ensuring transparency and accountability. This study of CRPs contributes to this process.

### Who and what are CRPs?

A CRP is a research professional employed to deliver clinical research. Unlike laboratory technicians or data managers, CRPs occupy roles that involve direct contact with people who are both patients and research subjects. CRPs work within hospitals and other healthcare environments and their primary objective is to facilitate the production, collection and management of data within clinical trials and other clinical studies. While they work directly with patients, they are not involved in delivering care outside the research pathway. In many respects, the role of the CRP and that of the CRN have many similarities. Indeed, many job listings advertise for a “*research nurse/clinical research practitioner*,” highlighting the blurred boundary between these two staff groups. According to the NIHR, around a quarter of the workforce funded by the NIHR Clinical Research Network are now working in CRP roles [25].

The CRP designation is a recent one, but the presence of ‘non-nurses’ working in clinical trials goes back decades. Drawing on a study conducted in the late 1990s, Mueller describes the seven types of occupational workers she observed as being involved in the day-to-day management of clinical trials [26]. Included on this list is a category she calls ‘Data Managers’ described as “*support personnel*” primarily engaged with tasks such as “*organising trial materials, maintaining clinical research records, updating and monitoring patient databases, ordering study medication, responding to sponsor initiated queries, scheduling and coordinating patient appointments, and readying the site for sponsor monitoring visits*” [26]. Notably, Mueller observed that “*on occasion data managers interacted with patients, assuming some of what nurses referred to as routine protocol appointments*”. Since then, what was once an ad hoc role – what our informant CRN-04 described as a “*pick-up-everything-else*” kind of job – has developed and become increasingly common within the research delivery process.

There are now efforts to professionalise the CRP role and establish it as a formal career. In 2017, the NIHR launched a task force aimed at systematising what had, up to that point, been an unplanned and inconsistent workforce. A directory was set up in September 2018, adopting the title ‘clinical research practitioner’ to include all patient-facing research staff not currently clinically registered. The directory is intended to “*create a Community of Practice as a foundational step in defining the professional identity of this diverse group*” [27] and to “*build a professional identity among Clinical Research Practitioners so they can receive relevant updates and speak with one voice*” [28]. The directory is currently voluntary but is intended as a first step towards professional registration through the Academy of Health Care Science and the roll out of a formal CRP qualification is planned in the future. The category of CRP is clearly a work in progress. However, the end point is the creation of a new career in research delivery that is changing the research system in the United Kingdom.

### Methods

The study was conducted in a large inner-city teaching hospital (acute trust) in London, United Kingdom. The hospital is host to a Biomedical Research Centre (BRC) – an infrastructural investment supported by the NIHR that joins hospitals with university partners to “*conduct translational research to transform scientific breakthroughs into life-saving treatments for patients*” [4]. This BRC supports a diverse portfolio of research that takes place across the trust’s many clinical directorates as well as its dedicated Clinical Research Facility.

The data we draw on in this paper are part of the BRC’s ongoing efforts to understand the evolving research workforce at the trust. In its mission to continue to develop and expand research activity, the BRC initiated an internal review of research delivery staffing in 2017. Building on two previous censuses, the BRC conducted a census in 2017 of all research delivery staff at the acute trust. Unlike earlier surveys that had included only research nurses, the 2017 census included the wider research delivery workforce. This is because research managers had begun to take note of the increasing numbers of individuals without clinically registered status working in research delivery across the trust. The 2017 workforce census was circulated to staff working within research settings at the trust; 581 people were invited via email to complete the survey and 298 responded, leading to a response rate of 51%. In conjunction with the census, the BRC also commissioned an in-depth qualitative study of the research workforce at the trust, the results of which we report on here.

Data were collected between September and December 2017. We used responses to the 2017 census to identify potential interview subjects. Candidates were selected in order to provide a range in terms of job role and clinical area. Of those who responded to the census, we excluded staff who indicated that they did not have patient-facing roles. Candidates were contacted by email. The sample was composed of CRNs, CRPs, Research Managers and Research Directors (RDs); 16 CRNs were invited to interview, of whom 8 representing 8 different departments accepted; 32 CRPs were invited to interview, representing 12 different job titles and 15 different departments. Of these, 10 accepted, representing 6 different job titles and 6 different departments. In the category of Research Managers and RDs, 10 people were contacted across 6 directorates, with 7 agreeing to participate, representing 3 directorates. In total, 25 staff were interviewed across 11 different directorates. Data collection was semi-structured with an overall focus on research staff backgrounds and roles, as well as their perceptions and experiences of careers in research. Interviews were an average length of 1 h and were audio recorded and transcribed. The data were managed using NVivo 11 software and analysed thematically, moving iteratively from descriptive coding through to themes, concepts and application of theory [29].

## Results

Our study of CRPs is organised around three themes:

1. Background, employment history, education and training
2. Work-related tasks, roles and relationships
3. Research delivery and the production of scientific knowledge

Here, we present the results of our study, focusing on the causes and consequences of the changing NHS research workforce, how CRPs are trained for their roles, and the challenges and opportunities CRPs represent to the research system.

### The push and pull of a changing workforce

At the trust where we gathered our data, CRPs are now the fastest growing segment of the research workforce. As the overall research workforce at the trust has increased over the past several years, the proportion of non-registrant CRPs has expanded relative to nurses. One factor influencing the increase in CRPs is the different levels of motivation between CRNs and CRPs around pursuing careers in research. CRNs in our sample described not being aware of research nursing as a potential career specialisation, or even having negative preconceptions of the role until ‘stumbling’ into it. CRPs, on the other hand, were more intentional in their pursuit of careers in research. Many were drawn to clinical research because it represented an opportunity to be involved in the research enterprise whilst also being able to interact with patients.

“*I went and worked in cancer for a year. It opened my eyes even more as to okay, I really, really enjoy this. I really want to make a difference to prolong life, find better treatments for people. It wasn’t just the patients this time around, it was also the carers, their families. We were having one to ones, you form a relationship.*” CRP-09

While there are technical or administrative roles in research that do not require contact with patients, the specific combination of research work in clinical trials and patient contact makes the CRP role particularly attractive. In effect, it offers ‘the best of both worlds’ and is the most commonly cited motivator among the CRPs we spoke to.

Another important factor accounting for the rise of CRPs within research is the ongoing pressure on the nursing workforce. Though in high demand in research, managers describe difficulties recruiting nurses into the role. As a result, CRPs are increasingly relied upon to fill gaps on study teams left open by the shortage of nurses; for example:

“*If I put* [an ad for a] *Band 6 research nurse, depending on the area, I would probably get between three and eight applicants. Maybe one, if I’m really lucky, will have prior research experience. If I put a Band 6 coordinator post out, in a week, I will have over 300 applicants. They will all be graduates, all with firsts or 2/1s. Most of them will have some kind of research experience. And most of them will have three or four years’ previous work experience.*” RD-01

The relative difficulty of recruiting qualified nurses is linked to the increasing proportion of CRP staff working in clinical research at the trust because research managers are unable to attract and retain nurses from the finite and currently stretched nursing labour supply. Managers have thus been pushed to recruit from the much wider pool from which CRPs are drawn in order to meet the staffing requirements of research delivery at the trust.

There is a larger pool of applicants for CRP positions because this role is open to a wider population with diverse educational and career backgrounds (Table 2). Instead of simply drawing on one qualification (nursing), managers are increasingly turning to recent graduates in an array of disciplines. In line with the results of the 2017 staff census, which found that all CRPs at the acute trust had first degrees, all the CRPs in our sample had degree qualifications, though there was wide variety in terms of field of study. Four had degrees in a clinical area, including midwifery, optometry, dietetics and sports science, four had degrees in scientific disciplines, including pharmaceutical science, neuroscience, psychology and chemistry, and two CRPs had first degrees in law.

**Table 2:** Clinical Research Practitioner sample

Degree		Prior experience		Time in post	
Clinical	4	Clinical	5	< 1 year	3
Science	4	Research	5	1 to 2 years	5
Other	2	Administration	3	3 to 4 years	1
				> 5 years	1

In addition to the educational diversity among CRPs, this staff group also come to their roles with a variety of previous work experiences, including working within the NHS in non-patient facing administrative roles, overseas clinical work, and research experience in the private sector. CRP-10 arrived in clinical research with no previous clinical experience, but many years of education and work history in both scientific research and data analysis.

“*I did chemistry at uni. I did a BSc and then I did a research masters. But my research masters was not pharmaceutical-based. But I think that still helps because it’s all broad research in general. It all kind of links up. And obviously the chemistry background is really helpful with knowing about the drug and drug mechanisms and things like that … And then I applied for this job here and got it. And I think it was quite a good combination of having done chemistry, which helped the science. But then also, my job as an analyst actually really helped, because of all the data – we have to do a lot of data processing and things like that.*” CRP-10

CRP-10’s description of the winding path she took that landed her in research is one that is shared by many CRPs we spoke to. The details vary, but what CRPs have in common is the diversity of their experience. This stands in marked contrast with their CRN colleagues, who must all go through a common course of education and training to become a nurse. The growth of CRPs, then, represents the diversification of educational backgrounds for patient-facing staff within the health system. They bring skills, like data analysis or other research specialisations, that CRNs may lack. However, it also poses challenges. Managers cannot assume a common body of knowledge and skills, and the health research system cannot rely on the accountability that registered status confers.

### Training and roles

The transformation from a science graduate or a solicitor into a clinical research delivery professional takes place on the job. CRPs, like all research workers employed in the trust, are required to undertake Good Clinical Practice (GCP) training [30], which provides an introduction to the ethical regulations and data management requirements that govern clinical trials. It also covers many aspects of the basics of clinical research delivery, such as the requirement to record an unexpected adverse event or ensuring that trial participants are properly consented. However, it does not cover the practicalities of the clinical, clerical and administrative duties research workers encounter day-to-day. For this, both CRNs and CRPs rely on the on-the-job training provided by study sponsors or their local study teams on study-specific procedures and protocol.

In addition to learning to read and interpret study protocols, patient-facing research staff undergo some level of clinical training and/or competency assessment. For CRNs, this usually means upgrading specific clinical skills necessary for their particular area of research – for example, skills related to peripherally inserted central catheter (PICC) lines for nurses working on oncology trials, or being signed off to perform the basic suite of clinical tasks that are associated with research, such as venepuncture, cannulation or electrocardiograms (ECGs). CRPs are also trained in similar clinical skills on the job but, unlike CRNs, they do not all have previous clinical training or experience in clinical settings to build on.

For those CRPs with no prior clinical experience, the process of acquiring basic clinical competencies represents a challenge, one that they variously described as “*stressful*” and “*daunting*”.

“*This is the first role that I’ve had that I need to be in clinic. I started this time last year. And I came with no clinical background at all. And then, all of that training was on the job. So, it’s vitals and ECG taking and venepuncture, cannulation. But, prior to that, I’d had no experience of ever having taken a patient’s bloods or cannulating patients or any sort of patient observation at all … I think, in the initial job spec, it wasn’t clear to me the level of clinical work that would be involved within the job. And, initially, I personally felt a little nervous. But the training that I received and the support that I got from my team was amazing.*” CRP-08

Though anxious about certain aspects of her new role, CRP-08 felt satisfied with the level of mentorship and support she was offered by a senior nurse colleague, a perspective shared by most of the CRPs in the sample.

On the other end of the spectrum, some CRPs arrive in research with extensive clinical training and experience, but their non-registrant status prevents them from utilising their skills due to what they see as arbitrary bureaucratic reasons. This can be frustrating or demotivating, as CRP-02 describes:

“*Where I used to work we had to do ECGs on clients, and read the ECGs. So, try and analyse the ECGs—are they regular, irregular? We had training on that, and had to go through practical exams with cardiologists. So it was quite thorough. And over here, we obviously have competencies that anyone wants. I offered myself to do ECG training because of my previous background. I’ve got an interest in it. I was told I can’t do it because I’m not a nurse. And I said, ‘Listen, I may not be a nurse but I feel that I’ve got the knowledge, more knowledge than some of the nurses around here in terms of this.’ And still it was like, ‘No you can’t do it.’ It was just like, ‘we do it’. When newcomers come in we have to train them on ECGs. To be honest this training wasn’t even how to read an ECG strip. It was how to place the electrodes. So, you’re saying that I’m not competent enough to tell someone else what I’ve been trained to do. So I thought that was quite demotivating actually.*” CRP-02

CRP-02 is describing a mismatch between his skills and the limits of his professional role. His frustration partly reflects the manner in which research labour is organised within the health service, where authorisation to perform particular tasks is tied to professional categories and job descriptions rather than to individual competencies.

In addition to acquiring clinical skills, research workers are also required to learn the administrative and data management systems necessary for work in research delivery. Even for those CRPs who may have had prior clinical experience, assimilating the range of knowledge and skills required in research can be challenging, as demonstrated by the experience of CRP-04, a former healthcare assistant.

“[Training in] *the NHS is very much on the job. I came from doing clinic to the research role, because it’s very similar – I knew how to take blood and I knew how to do some nursing skills. Things like taking blood pressure and simple things like that. But it was a completely different set of learning to learn about all the ethical issues and research values and CRFs and all these abbreviations and copy here and copy there … I mean the GCP training, everything went so quickly, it was two hours. And you go through all those different topics. But, for them* [CRNs] *to just live it every day, you know, to be so accurate with the data and to understand the knowledge of protocols and SOPs* [standard operating procedures] *and all these things related to – I don’t know, it’s just a different world, isn’t it, research. I cannot even explain it properly.*” CRP-04

The on-the-job method by which research workers acquire both clinical and research competencies is standard for all, and largely follows methods established by the trust. However, the content of the competencies varies by division and by study. At the time of writing, there was no core set of competencies for all CRPs. Additionally, beyond GCP, there are no research-specific training materials to help them acquire the particular skills or competencies they will need in their jobs. Access to research-specific training varies across departments, with some research workers benefiting from large, well-organised research teams with access to qualified supervisors, mentors and established training programmes. Other research workers, especially those working in isolation, can find themselves without access to appropriate training, supervision or support. In CRP-04’s case, she was required to complete a training booklet that was not relevant to her role, and was unable to find research-specific training materials, even when she requested them.

“*I think training in research is a bit more difficult to come by. The other day, they send me these healthcare assistants’ booklet and it was nothing related to research … The questions were for healthcare assistants working on the ward. Like, what’s the importance of food rounds? I don’t do food rounds. Or night shift. I am supposed to learn something from it? I found it very pointless for people in my job role.*” CRP-04

While CRP-04’s experience was not common to all CRPs, her frustration with the lack of research-specific training highlights a challenge faced by many. In the absence of a standardised core set of training courses or materials in both clinical and administrative competencies, some CRPs feel that their roles are not well defined and not well understood. Further, some research managers feel concerned about the potential consequences of the lack of transparency and standardisation in CRP training for both patient safety and the validity of clinical data collected, as we discuss below.

The lack of standardised training carries through to a lack of standardised roles for CRPs. On the job, clinical research involves a wide range of tasks and responsibilities, though this is not always clear to patients, study principal investigators or other hospital staff. CRNs and CRPs often undertake very similar and overlapping roles, which can blur the boundaries between these two staff groups. As a result, supervisors see the standardisation of the role of the CRP as an important goal. CRN-04 oversees teams that include both CRNs and CRPs:

“*We’ve been doing a lot of work around it because it wasn’t really standardised five years ago. It was just a coordinator-picked-up-everything-else kind of job. So we worked hard on creating the same sort of competencies that a nurse would have. So, you have objectives. You’re assessed three or four times with an appropriate person and then you have a proper assessment at the end. I think something like that, it’s beneficial to everyone. It’s beneficial to whoever’s line managing them to see actually where they are. But it’s beneficial to the coordinators and practitioners as well, to actually see what it is that’s expected of them* […] *For all new people coming in, they’ll get a competency document in the same way a student nurse gets a competency document and they work their way through, just to make sure that everyone’s on the same page. And then it’s good as well because obviously it’s encouraging* [band] *6s to look at their team and go ‘what actually are people doing in the team? What are they responsible for?’ Just knowing what people’s boundaries are. I think it’s really important because, at the moment it’s a bit fluid.*” CRN-04

For CRN-04, basing CRP competencies on what a “*nurse would have*” ensures that all research staff are equally skilled in aspects of clinical practice and addresses the somewhat “*fluid*” reality of research work. CRP-03, a CRP with the Clinical Research Network, underscores the blurriness in the boundaries between CRN and CRP roles in clinical research:

“*There’s certainly a bit of confusion. I was under the impression until quite recently –and I was told in my previous roles – that the RCN* [Royal College of Nursing] *research nurse competencies could be used for non-nurses. But then I was told, really not that long ago, in the last year or so, that that’s not the case…. I certainly think that there ought to be some standardisation in the competencies. Obviously, I appreciate that there are some things that a nurse can do that a practitioner can’t, so there would be some minor differences. But for the most part, they would be the same.*” CRP-03

CRP-03’s experience illustrates the uncertainty many CRPs have about the scope of their roles, and the extent to which the CRP and CRN staff groups have, in some places, been deployed interchangeably. The lack of specific training or standardised roles for CRPs is a result of their relatively recent entry into the hospital workforce. At time of writing, the acute trust was developing training materials, curriculums and competency frameworks to standardise job descriptions and put in place a management and oversight structure for CRPs. This effort underscores the growing importance of CRPs and the need to think carefully about patient safety and research outcomes within the changing clinical research landscape.

### Challenges and opportunities of a changing workforce

The transformation of the research delivery workforce presents a number of challenges as well as opportunities to the conduct of research and the delivery of patient care within the research pathway in the NHS. As the numbers of CRPs have increased in the acute trust, so has the uneasiness of senior managers around questions of safeguarding patient safety.

The need for standardised roles is clear, as some CRPs have been asked to perform tasks in which they are not competent or qualified to take on. In part, this may evince a lack of understanding on the part of doctors and principal investigators. CRP-08 provides an example:

“*You’ve been asked to do this by the doctor, but really, that’s not the responsibility of the CRP. I think the difficulty is, when you’ve been asked by somebody who you see as being high up in the hierarchy, to do something that they feel you’re qualified to do, and it’s right for you to be doing that thing.* [ … ] *In our last meeting we talked about appropriate escalation and taking on tasks appropriate to your role and being aware that things may not be appropriate even if a doctor’s asking you. But interestingly, there is no guidance as to what is appropriate. So just being told, ‘Oh just always make sure that you’re doing things appropriate to your role.’ But, what is that, I don’t know.*” CRP-08

As CRP-08 points out, in some cases, CRPs are unsure about the boundaries of their practice and feel that they lack the ability to say no to someone ‘high up in the hierarchy’. Though her team are addressing the issue through meetings and discussion, she points to a lack of “*guidance as to what is appropriate*”. In certain instances, this can cause frustration for CRPs and their colleagues.

The lack of clarity concerning the relationship between research and patient care in the professional remit of CRPs can also cause frustration for patients. For example, to some patients, CRPs look like nurses, yet they are not nurses and are not authorised to perform nursing tasks. CRN-04 describes how this situation can arise:

“*We have CRPs who can access PICC lines. But again, they go through the same competency document* [as a nurse would]*. But yes, we do have a couple of practitioners that are skilled and they’re competent in the same way that a nurse would be. The only difference I guess, is I do feel a lot of the time, with practitioners, you give them half the role, but not the full job. So, yes, they can access a PICC line. You’re saying they’re competent at doing that, but then if the PICC line becomes blocked, they can’t flush any medication down to unblock it. So, they’re kind of halfway houses and then they have to go and get nurses and then I think, well actually is it worthwhile giving them half the job? Either let them do the job or don’t let them do the job. Because they go up to see the patient to take the blood, the PICC blocks, they have to find a nurse, they need to wait an hour for a nurse to appear from somewhere, while the whole time they’re patient facing and the patients are like, ‘Why can’t you just do it? You access the bloods so just put the medication down.’ We’re giving them half a job.*” CRN-04

Here, CRN-04 highlights a dilemma surrounding CRPs at work. In many cases, they are assessed as competent and given the roles, responsibilities and, crucially for patients, the appearance of highly skilled and trained clinical research nurses. However, they lack the formal, regulatory capacity to function as nurses. CRP-09 describes this situation succinctly:

“*I am now a practitioner and I call myself almost a nurse, but I’m not a nurse. Because obviously I can’t prescribe, you know sign off medicine and things like that. But everything else a nurse can do, I can do it…. I introduce myself as I’m like a nurse but I’m not a nurse.*” CRP-09

CRPs are “*like a nurse but not a nurse*” in that their job requires them to perform nursing tasks, and they report patients seeing them as nurses, and yet in important official ways, they are not nurses and are unauthorised to fully adopt the nursing role. It is clear that this ambiguity is not incidental to the clinical research process. It has been produced by the specific ways in which the CRP role evolved.

The frustration experienced by research workers – and, as reported by these workers, experienced by patients as well – is a consequence of the lack of clear guidelines of practice for CRPs, or boundaries around the scope of their role. It emerged as a “*pick up everything else*” kind of job and has been adapted to fit the needs and requirements of the various study teams and directorates where CRPs are employed. As their numbers have increased, so too has the variety of the work they can be found doing. Yet, without standard role delineations, it is difficult for CRPs, and their patients and managers, to know what they can and cannot do.

The hybrid professional identity of CRPs represents, for many interviewees, one of the attractions of the job. They value the opportunity to conduct research, but equally value being able to conduct this research through interaction with patients. However, this hybrid role is uncodified, and the lack of clarity regarding what they are and are not authorised to carry out in clinical settings can be a source of frustration. As the research workforce within the NHS grows and the CRP role is expanded and standardised, the challenge will be finding ways to preserve the flexibility of the CRP workforce – upon which the clinical research process has come to depend – but at the same time avoid the frustration and inefficiency that stems from the lack of clear definition and undefined expectations for this crucial role.

## Discussion

The CRP role is being developed in response to the creation of a health research system within the NHS. The CRP is emerging as an organisational solution to the question of how to deliver clinical research against the backdrop of a decreasing national nursing workforce. With current pressures on the nursing workforce and the loss of bursaries for nursing undergraduates, the nursing workforce challenges are set to continue, which suggests that the CRP workforce is likely to continue to grow. This paper has sought to understand the ways in which this role differs from CRNs and to explore what this role and professional identity means for those who occupy it. Our data broadly suggest that the work of CRPs overlaps significantly though not completely with the work of CRNs, but that the CRP role lacks a clear definition and scope of practice.

The growth of CRPs within the health service reflects a number of needs and goals, but primarily it has been driven by the need for staff to handle increasing volumes of clinical research amidst the context of a shortage of nurses. This need has not been met by simply growing the research workforce; it has meant a change in the composition of the workforce itself, represented by the growth of CRPs relative to CRNs. Our data suggest that this presents a number of challenges, as CRPs are not authorised to perform certain tasks, cannot be assumed to possess certain competencies and may require different sorts of on-the-job training. However, our research also suggests that the growth and professionalisation of the CRP role is also an opportunity, as these workers bring a diversity of educational and professional backgrounds to the research delivery process and possess a wide set of skills.

If the growth of the CRP role does represent an opportunity for the health research system, our data suggest some specific ways in which this opportunity can be best maximised. Many of our informants would like to see more clarity regarding the roles and responsibilities of CRPs. Many nurses and CRPs alike feel that organisations should develop consistent and clearly defined professional roles for their practitioner workforce with a view to ultimately achieving consistency across the health service. Our interviewees would like to see more progress towards professionalising the CRP role. Many informants would also like to see a clearer picture of the CRP as a career. Our informants feel limited in their career progression owing to limits to their practice and the lack of availability of senior roles; several indicated that they planned to leave the NHS in order to pursue career advancement. This potential attrition represents a drain on resources for the NHS, and a missed opportunity to develop and encourage a skilled and motivated workforce. In response, we should recommend that administrators and planners within the NHS seek to provide a clearer picture of the CRP career ladder.

Many of the challenges described by our informants reflect the fact that CRPs are undergoing professionalisation but that the process is far from complete. There is currently a directory for CRPs, but it is underdeveloped compared to the register for nurses and other healthcare professions. The CRP directory has no regulatory mechanisms, no enforcement powers and no code of conduct, though all of these could be added in the future. There is a need for professional clarity and regularity that so far has been unmet. Furthermore, developing a directory and other signs of professionalisation may not be a way to address broader changes in medical research and care raised by the emergence of the CRP. While creating a CRP directory, and ultimately a registry, is aimed at allaying concerns expressed by some respondents around issues of responsibility, accountability and visibility of CRPs in hospital-based clinical research, the question remains regarding what is lost and what is gained when certain tasks, once performed by nurses with clinical training, experience and judgement, are undertaken by non-nurses. Even when CRPs have been more fully professionalised, their relationships with nurses and other professional roles and broader questions regarding the division of labour within healthcare will still need to be negotiated and clarified.

The rise of the CRP, as we have said, represents challenges as well as opportunities. Future research might examine how the health service can harness the enthusiasm of people who choose to work as CRPs, as well as how it might manage the ambiguities that attend to their roles. How can the health service ensure patient safety while at the same time supporting CRPs in their interactions with patients and research participants? There is a need for research examining how patients interact with CRPs as well as other research workers.

Future research might also explore, in more general terms, the consequences of the growth of health research workers without clinical backgrounds. What does the growth of CRPs mean for CRNs – will it lead to a hierarchical relationship between clinical and non-clinical researchers? How are crucial clinical responsibilities and caring work divided amongst health research professionals, and how does this process of professional change shape research outcomes, career possibilities and the patient experience? Furthermore, the nascent professionalisation of CRPs may be part of a broader trend within the health service, which is seeing the professionalisation of various roles such as paramedics [31], pharmacists [32] and occupational therapists [33]. How extensive is this pattern, and does it imply the decentring of doctors and a change in the ways in which patients interact with the health system?

The study has a number of limitations. First, as an interview-based, qualitative study, we sought to explore perceptions, motivations, opinions and experiences from the perspective of clinical research staff as they were represented to us. We have aimed at representing this perspective in a valid way, but we make no claims to statistical generalisability. Second, our findings are based upon interviews with research staff themselves, and therefore should not be treated as providing a picture of how the emphasis upon research is changing the NHS as a whole. We did not interview patients, administrators, outside experts, policy-makers or the general public, all of whom can be seen to be either shaping the role of research within the NHS, impacted by it or both.

## Conclusion

This study is a close examination of the clinical research workforce at one research-intensive acute trust in the United Kingdom, with a focus on the emerging CRP staff group. Drawing on semi-structured interviews with clinical practitioners and other clinical research staff, we show that CRPs are drawn from a wider pool of educational and employment backgrounds than their CRN colleagues, and actively seek out work in health research. CRPs receive on-the-job training to acquire the competencies that are necessary for them to carry out their work. We also show how the CRP role, which began in an ad hoc manner, has become regularised. However, the CRP role is still not well defined, which can be a source of frustration for CRPs and those who work with them. At the same time, the professionalisation of the CRP workforce represents an opportunity for the health research system to employ research workers who possess a range of in-demand skills and to shore up research capacity in the context of the shortage of nurses.

Understanding the evolving role of CRPs within research delivery can help shed light on how a changing health system is driving the demand for new forms of labour, forging new careers for research workers and creating new connections to industry. It can also contribute to a better understanding of the organisational factors and social processes that enable the production of health research and medical knowledge.

## Data Availability

The data generated and analysed during the current study are not publicly available due their containing information that could compromise research participant privacy but are available from the corresponding author on reasonable request.

## Abbreviations

BRC: Biomedical Research Centre
CRN: Clinical Research Nurse
CRP: Clinical Research Practitioner
ECG: electrocardiogram
GCP: Good Clinical Practice
NHS: National Health Service
NIHR: National Institute for Health Research
PICC: peripherally inserted central catheter
RD: Research Director
